# What Is the Effectiveness of Different Duration Interdisciplinary Treatment Programs in Patients with Chronic Pain? A Large-Scale Longitudinal Register Study

**DOI:** 10.3390/jcm9092788

**Published:** 2020-08-29

**Authors:** Elena Tseli, Riccardo LoMartire, Linda Vixner, Wilhelmus Johannes Andreas Grooten, Björn Gerdle, Björn O. Äng

**Affiliations:** 1 Department of Neurobiology, Care Sciences and Society, Division of Physiotherapy, Karolinska Institutet, S-141 52 Huddinge, Sweden; riccardo.lo.martire@ki.se (R.L.); wim.grooten@ki.se (W.J.A.G.); bja@du.se (B.O.Ä.); 2 School of Education, Health and Social Studies, Dalarna University, S-791 88 Falun, Sweden; lvi@du.se; 3 Functional Area Occupational Therapy & Physiotherapy, Allied Health Professionals Function, Karolinska University Hospital, S-171 76 Stockholm, Sweden; 4 Pain and Rehabilitation Centre, and Department of Health, Medicine and Caring Sciences, Linköping University, S-581 83 Linköping, Sweden; bjorn.gerdle@liu.se; 5 Center for Clinical Research Dalarna-Uppsala University, S-791 82 Falun, Sweden

**Keywords:** multidisciplinary pain management, outcome measures, physical and mental functioning, practice-based evidence, registry study

## Abstract

Chronic pain is a leading cause of disability globally. Interdisciplinary multimodal pain rehabilitation (IMPR) targets pain with a bio-psycho-social approach, often delivered as composite programs. However, evidence of optimal program duration for the rehabilitation to succeed remains scarce. This study evaluated the effectiveness of different duration IMPR-programs—using within- and between-effects analyses in a pragmatic multicenter register-based controlled design. Using the Swedish Quality Registry for Pain Rehabilitation, data from fifteen clinics specialized in chronic pain rehabilitation across Sweden were retrieved. Participants were patients with chronic musculoskeletal pain who had taken part in short (4–9 weeks; *n* = 924), moderate (10 weeks; *n* = 1379), or long (11–18 weeks; *n* = 395) IMPR programs. Longitudinal patient-reported outcome data were assessed at baseline, post-intervention, and at a 12-month follow-up. Primary outcomes were health-related quality of life, presented as perceived physical and mental health (SF-36). Secondary outcomes included the Hospital Anxiety and Depression Scale (HADS), pain intensity (NRS 0-10), the Multidimensional Pain Inventory (MPI), and perceived health (EQ-5D). Overall, all groups showed improvements. No clinically important effect emerged for different duration IMPR. In conclusion, while our results showed that patients following IMPR report improvement across a bio-psycho-social specter, a longer program duration was no more effective than a shorter one.

## 1. Introduction

Chronic pain, typically defined as pain lasting ≥3 months or beyond the point of normal tissue healing [1], is highly prevalent all over the world and constitutes one of today’s leading public health challenges [2]. Furthermore, chronic pain is often accompanied by a distinctive bio-psycho-social complexity; it negatively affects physical and emotional functioning, social interaction, quality of life, and work ability-resulting in poor overall health [3,4] and disability, as reported in the 2017 Global Burden of Disease study [5].

Interdisciplinary multimodal pain rehabilitation (IMPR), also known as interdisciplinary treatment [6], is considered a core intervention for mitigating chronic pain. In terms of decreasing pain and disability, evidence shows that a multicomponent and multidisciplinary approach is superior to single-treatment measures [7,8,9,10]. The IMPR approach adopts a bio-psycho-social perspective and, using the principles of behavioral therapy as a foundation, it incorporates a combination of physical activity/exercise, psychological measures, pharmaceutical treatments, and patient education; all administered by an IMPR team [11] that includes physicians, physiotherapists, occupational therapists, and other health professionals, working closely together using a “shared biopsychosocial model and goals” [6].

Providing high-quality research on IMPR programs has been recognized as a major challenge due to the complexity of the treated conditions and diverse nature of IMPR elements [12]. Systematic reviews have shown multidisciplinary treatments to be effective [7,8,9,10] but comparisons on the optimal composition, intensity, and duration of IMPR programs remain inconclusive [13]. There is presently a paucity in scientific literature on what treatment lengths are effective; a first-order prerequisite for informed and improved healthcare delivery. IMPR duration, as one dimension of dosage, is certainly complex but the topic has been proposed as a priority research area in pain rehabilitation networks and patient associations. From the context of social cognitive theory, principles of behavior change imply that behavioral changes require time to learn and practice, and therefore interventions should provide sufficient time to adopt these skills into daily life [14,15,16]. However, until now, there has been no available evidence of treatment duration having an influence on the success of the intervention [9]; variations in treatment time between clinical centers are essentially based on local tradition and medical staff preferences—which is the situation currently seen in Sweden.

Pragmatic clinical registry studies are currently promoted for establishing clinical evidence relevant to real-world practice [17,18]. At present, IMPR programs are offered at roughly forty specialist clinics across Sweden, all of which are linked to the Swedish Quality Registry for Pain Rehabilitation (SQRP). The SQRP aggregates data from a comprehensive set of patient-reported outcome measures (PROM), following current recommendations [19,20]. The present longitudinal study sought to evaluate the effectiveness of moderate and long duration IMPR in patients with chronic pain, compared to a reference group receiving short duration IMPR. Based on our clinical and theoretical understanding, along with previous research [8,21,22], our hypothesis was that IMPR programs would have positive effects on our defined outcomes and that a longer duration IMPR program would be superior to a shorter duration IMPR program.

## 2. Materials and Methods

### 2.1. Design

This pragmatic superiority-controlled trial adopts a longitudinal, multicenter, registry study approach. PROMs were obtained from the SQRP representing 15 outpatient specialist clinics in Sweden that provided reliable data for delimitating between short (4–9 weeks; 6 clinics) vs. moderate (10 weeks; 4 clinics) vs. long (11–18 weeks; 5 clinics) IMPR programs. The cut-off in duration-time, here defining our independent intervention groups, was selected to get three well-delimited and pragmatic groups of different program duration, based on how modern IMPR is currently applied. The project has been granted ethical approval (EPN Stockholm, reference number: 2013/1842-31/2). Written informed consent forms were collected from all participants. All participation including self-ratings on questionnaires was voluntary and is routinely collected in the SQRP database as standard procedure, so did not add to the workload of either patients or healthcare personnel. The study protocol is registered at ClinicalTrials.gov (id: NCT02248363). Public and Patient Involvement statement: Patients and the public were not involved in the design and conduct of the study.

### 2.2. Participants

A total of 2698 patients were referred to IMPR specialist clinics for evaluation, and subsequently were selected for IMPR programs during the period 2012–2016. Data covered baseline measures (patient characteristics and outcomes), and subsequently followed up outcome measures post-intervention and at a 12-month follow-up. Inclusion criteria were: Patients aged between 18–67 years (i.e., working-age population) with disabling chronic musculoskeletal pain, defining chronic as a duration of ≥3 months, and delimitating musculoskeletal pain conditions to benign, nonspecific, pain diagnoses such as back or neck pain and fibromyalgia or general widespread pain but not including pain emanating from, for example, systemic or inflammatory diseases (e.g., rheumatoid arthritis), or degenerative joint diseases (e.g., osteoarthritis-related joint pain) or malignancies. A confirmed consent to be registered in the SQPR was also set as mandatory for inclusion in the study. Patients were referred from primary care, occupational health clinics, and other clinics within hospitals. They were initially assessed by an interdisciplinary team through interviews and examinations, and a rehabilitation plan was formed in agreement with the patient.

### 2.3. Intervention

IMPR programs were provided by interdisciplinary teams specializing in pain management. In accordance with the Swedish Association of Local Authorities and Regions national medical guidelines for IMPR in chronic pain, specialist pain management should be delivered by IMPR teams consisting of at least three professions; a physician specializing in rehabilitation medicine, a physiotherapist, a social worker, and a psychologist, plus an occupational therapist if needed [23]. In Sweden, IMPR programs are group-based, with groups usually consisting of six to eight participants. Within these groups, each patient has an individual plan, schedule, and individual contacts, according to the patient’s own goal setting.

IMPR was conveyed from cognitive behavioral therapy (CBT) perspective, typically involving psychological measures such as CBT (including acceptance and commitment therapy (ACT)), physical exercise and activity training, and patient education on chronic pain (as well as pharmaceutical treatments, when needed). Details on the composition of each clinic’s IMPR program are presently not reported in the SQRP, however, when we look to IMPR programs internationally, some variations exist in practice. Similarly, with regards to treatment duration, IMPR programs typically last 10 weeks, with a range of 4–18 weeks, with treatment delivered on average 3 days/week (range 2–5 days/week) and with a varying amount of hours/day. In general, programs were compound, either with a shorter duration and more days/hours per week or a longer duration and fewer days/hours per week. In total, all included IMPR programs consisted of ≥100 h of face-to-face contact between clinicians and patients, hence all included programs could be categorized as high-intensity interventions [8].

### 2.4. Outcome (Dependent) Measures

#### 2.4.1. Primary Outcome

Primary outcomes were health-related quality of life, presented as physical and mental functioning, post-intervention and at 12 months follow up. The Short-Form Health Survey (SF36), a self-assessed multi-purpose short-form health survey with 36 questions reflecting health-related quality of life, was used for this purpose. The 36 included questions yield an 8-scale profile of functional health and well-being scores, which can be incorporated into the psychometrically-based physical and mental health summary measures; SF-36 Physical Component Summary (SF-36 PCS) and SF-36 Mental Component Summary (SF-36 MCS). These two summary measures of SF-36, both ranging from 0–100, were used as dependent measures of physical and mental health status. The SF-36 has been proven useful in surveys of general and specific populations, for comparing the relative burden of diseases and in differentiating the health benefits produced by a wide range of different treatments [24].

#### 2.4.2. Secondary Outcomes

Self-rated pain intensity the past seven days was quantified with the 11-point Numeric Rating Scale, with 0 representing no pain and 10 representing the worst possible pain [25]. Perceived health status was assessed using the EuroQol5-dimensions (EQ-5D). This is a self-assessed standardized measure that provides a simple, generic measure of health for clinical and economic appraisal. It is applicable to a wide range of health conditions and treatments and it provides a single index value for health status (where a score of 1 represents “perfect health” and values below 0 represent states “worse than death”) that can be used in clinical and economic evaluations of health care as well as in population health surveys [26]. Further, and more specific to the experience of chronic pain, dimensions of pain intensity, emotional distress, cognitive and functional adaptation, and social support were assessed using the Multidimensional Pain Inventory (MPI) [27]. In accordance with the procedure described by McKillop et al., two summary scores were used for the presentation of domain impairment and social support, MPI impairment (0–6, where high scores represent high perceived impairment) and MPI social support (0–6, where high scores represent high social support) [28].

Finally, symptoms of anxiety and depression in patients with somatic diseases were assessed using the Hospital Anxiety and Depression Scale (HADS), which is a widely-used patient self-rated scale with 14 questions; 7 addressing “anxiety” and 7 addressing “depression” (subscales; HADS-A—anxiety and HADS-D—depression) that each ranges from 0–21 [29].

### 2.5. Independent Variable and Statistics

SQRP-data on IMPR duration (weeks) was used for the categorization of the independent variable duration into three groups, short (representing 4–9 weeks), moderate (10 weeks), and long (11–18 weeks) IMPR programs. In order to control the data quality for the current period (2012–2016), it was crosschecked against independent information from clinic representatives, which yielded 15 clinical settings with validated data on IMPR mean duration.

For each outcome defined, generalized estimating equation (GEE) with robust standard errors was used to compute the average effect over time within subjects, from baseline to rehabilitation end and one-year follow-up [30]. Standardization based on a general linear model with robust standard errors was used to compute the marginal effects between the duration groups (*short* vs. *moderate* vs. *long*) [31], adjusted for outcome baseline status. Correlations between patients’ baseline characteristics and the independent variable were used to ascertain that no additional variables confounded the analyses. Follow-up time was consistently used as within subject factor. GEE handle missing data by including all available data in the estimations. Significance was nominated at *p* < 0.01 for all analysis stages. Effect size Cohen’s d, a measure of an effect independent of sample size, was calculated to aid comparison with other studies; where effect-size values below 0.2 were considered small, 0.5 medium, and 0.8 large [32]. In addition, all outcomes were trichotomized into patients attaining a change equivalent to a minimal clinically important difference (MCID) in either a positive or a negative direction (improved or deteriorated) and patients who did not report an MCID change (no change) [33]. Here, for each subject, an increase of ≥3 for SF-36 (PCS and MCS) [34,35], ≥0.1 for EQ-5D index [36], and ≥0.6 for MPI Social support [37] were all regarded as an improved (or deteriorated) MCID. The same applied to a decrease of ≥2 for NRS [25,38], ≥0.6 for MPI impairment [37], and ≥1.5 for HADS-A and HADS-D [39]. Proportions of participants according to MCID were calculated from available data, i.e., reduced sample as per protocol analysis.

## 3. Results

**Figure 1:**
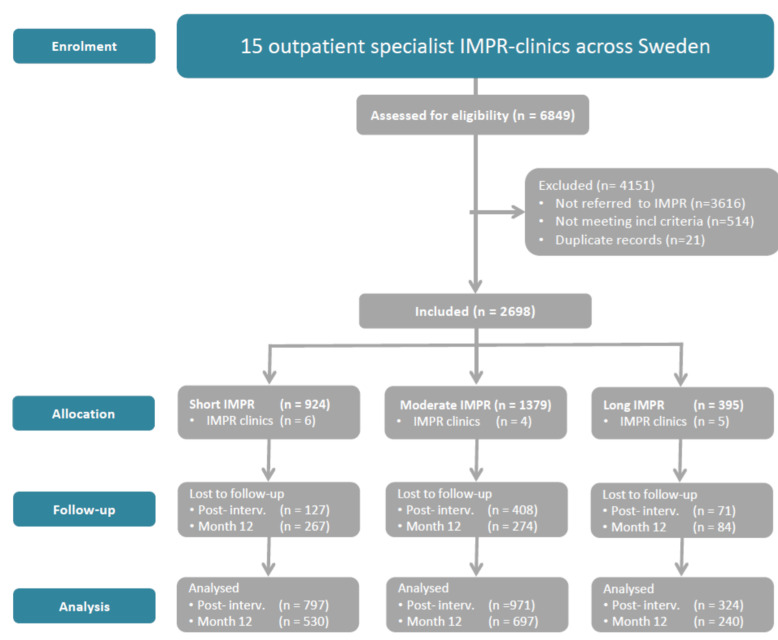
Flow chart of research design and patient participation, treatment duration and evaluation. SQRP: Data from the Swedish Quality Registry for Pain Rehabilitation. IMPR: Interdisciplinary multimodal pain rehabilitation.

The recruitment and retention of patients is shown in Figure 1.

In all, 2698 patients meeting inclusion criteria were referred to IMPR and completed an IMPR program, either a short (*n* = 924), moderate (*n* = 1379), or long (*n* = 395) program at one of the 15 specialist clinics. Of these, 2092 (78%) completed the post-intervention follow-up, and 1467 (54%) provided data at the 12-month follow-up (attrition rates calculated on primary measures).

Analyses on baseline data revealed no clinically important differences between participants that provided data at the 12-month follow-up vs. those missing. Subject characteristics for the three groups were similar (Table 1), and with similar proportions of prevalent pain diagnoses (back and neck pain and widespread pain). Regression analyses showed though that a longer duration was somewhat related to “treatment days/week” (r = −0.25), indicating that the longer duration groups have less treatment days/week. This variable was therefore adjusted for in all our between-group GEE-analyses.

### 3.1. Results of Main Outcomes

#### 3.1.1. Within-Group Analyses

Table 2 presents means and 95% CI, respectively, of the course in SF-36 physical and mental health from baseline to 12-month follow-up. Results showed that the patients improved significantly post-intervention, and that the improvement remained at the 12-month follow-up (*p* < 0.001). Regarding physical health, 41% improved significantly post-intervention according to MCID; however, 24% deteriorated on MCID. At 12-month follow-up, this was 44% and 22% respectively. Regarding mental health, the corresponding MCID for improvement/deterioration was 51%/26% post-intervention and 49%/28% at the 12-month follow-up.

#### 3.1.2. Between-Group Analyses

**Figure 2:**
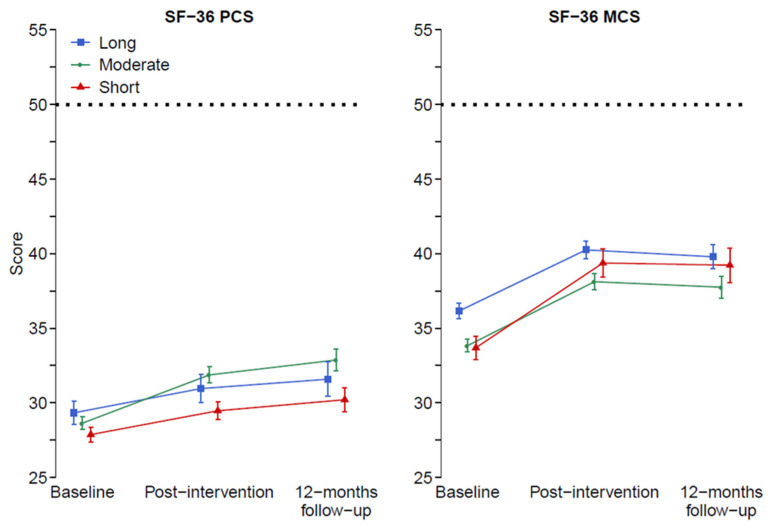
Change of health-related QoL from baseline to post intervention and 12 months follow-up, measured by SF-36 Physical Component Summary (PCS), Mental Component Summary (MCS). High values indicate better status. In the model, adjustment was made for baseline status and treatment days/week. Note: graphs show unadjusted baseline values, while statistical analysis was controlled for differences at baseline. Dashed lines indicate norm value for general population.

Table 3 and Figure 2 present mean (95% CI) modeled values, and non-modeled crude data, respectively, of the course in physical and mental health from baseline to 12-month follow-up. Using “treatment days/week” and baseline values as covariates, an effect emerged for the follow-up *group for physical heath, revealing the moderate-duration group improved more than the short-duration group, postintervention and at 12-month follow-up (*p* < 0.01). However, there was no effect for the long-duration group vs. short-duration group; results rather indicated that this group improved less than the short-duration group (*p* = 0.335 post-intervention). Regarding mental health, there were neither main nor any interaction effects from the follow-up course.

**Figure 3:**
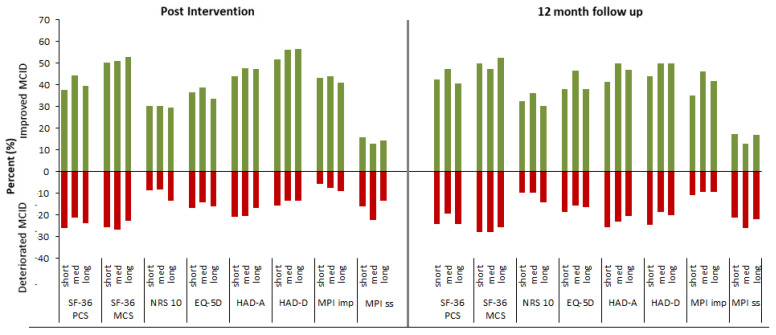
Figure illustrates minimal clinically important difference (MCID) for primary and secondary outcomes at post-intervention and 12-month follow up. Positive bars (green) show proportion of patients that improved while negative (red) shows proportion of patients that deteriorated.

Figure 3 shows proportions of subjects that improved or deteriorated, on MCID from baseline to 12-month follow-up (as per protocol). Results on MCID for physical function revealed no important differences between groups, revealing that about 40% (38–44%) improved post-intervention, while just over 20% deteriorated (21–26%). At 12-month follow-up, these percentages were 45% (42–47%) and 23% (20–24%), respectively. Regarding mental health, the corresponding MCID for improvement/deterioration was somewhat greater at just over 50% (50–53%) and 25% (23–27%) post-intervention, and again 50% (47–53%) and 27% (26–28%) at 12-month follow-up, respectively.

### 3.2. Results of Secondary Outcomes

#### 3.2.1. Within-Group Analyses

Table 2 presents means (95% CI) of the course in secondary outcomes from baseline to 12-month follow-up. Results showed that all treatment groups improved significantly for all secondary outcome measures post-intervention and remained at 12-month follow-up. The MCID for improvement/deterioration ranged from 13–56%/6–21% post-intervention and 13–50%/9–26% at 12-month follow-up. It was noted that the greatest MCID was on HAD D (depression) and MPI impairment, however, this was most evident post-intervention.

#### 3.2.2. Between-Group Analyses

Table 3 presents means (95% CI) of the course in secondary outcomes from baseline to 12-month follow-up. Using “treatment days/week” and baseline values as covariates, there were no between-group effects, main effects, nor any interaction effects for our secondary outcomes (NRS-10, EQ-5D, HADS-A, HADS-D, MPI-impairment, and MPI-Social support,) during the follow-up course. Results on MCID did not reveal any important differences between the groups (Figure 3).

## 4. Discussion

### 4.1. Summary of Main Findings

We sought to evaluate IMPR’s overall effectiveness and the comparative effectiveness of different durations of IMPR programs in patients with chronic pain. As a group, the results showed patients improved on our main outcomes, physical and mental health, at post-intervention and the effects were sustained at the 12-month follow-up. This result was also evident in our secondary outcomes, thus supporting our hypothesis that IMPR programs would have positive effects on our defined outcomes. Nevertheless, despite nearly half of the patients reaching an MCID improvement, there was a noticeable proportion of patients that clinically deteriorated on MCID during the follow-up period. IMPR proved equally effective irrespective of program duration, which contradicted our main hypothesis stating that longer IMPR programs are more effective.

### 4.2. Effectiveness of IMPR

Following an IMPR program, patients improved significantly post-intervention and the effects were sustained at the 12-month follow-up. According to MCID post-intervention, a larger proportion of patients improved in mental health than in physical health (50% vs. 40%), however, this difference was less at the 12-month follow-up (45% vs. 50%). This confirmed our hypothesis that patients would present an improvement over time, across a biopsychosocial specter.

Our findings are mostly consistent with several systematic reviews, which have concluded that IMPR generally is effective, but with small to moderate effects [7,8,9,10,13]. They are also in line with previous primary studies reporting long-term improvements on pain and quality of life [40,41]. In contrast, an umbrella review from 2018 assessing the strength of the evidence for the effectiveness of IMPR concluded that there is currently no robust evidence for the effectiveness of IMPR in any outcome, with the possible exception of decreased pain short term [42]. One major contribution to this conclusion was the small number of studies included in the synthesis and the fact that many studies were susceptible to small sample sizes. Our results are based on large, real-life empirical data, with width national representativeness.

Importantly, although our results showed positive effects at a group level, data on our MCID indicated that around 20% of the patients deteriorated at least one MCID on the primary and secondary outcomes, and that is despite receiving IMPR. This is in line with recent findings from Vartiainen et al. [43], who also showed that while health-related quality of life was improved in nearly half of the patients, there were also approx. 30% who reported a clinically important deterioration at 12-month follow-up after rehabilitation. Until now, research results that show good (or no) overall group effects have rarely shown subgroups which, paradoxically, may tend to deteriorate, possibly because they often lack power. It is believed that the identification of such subgroups has the potential to give important indications on how IMPR may be better tailored to each patient, and possibly also show which patient groups are in need of another type of rehabilitation. Further research presenting bidirectional data is thus warranted to increase knowledge of possible explanatory characteristics.

### 4.3. Comparative Effectiveness

Contrary to our hypothesis, IMPR proved equally effective irrespective of program duration. Longer IMPR programs were not superior to shorter programs and although there was indeed a statistically significant between-group effect favoring moderate vs. short programs, the difference was negligible and not detectable nor on MCID nor on our effect size calculation on Cohen’s d.

Our findings are, however, in agreement with the findings from systematic reviews that have examined the influence of treatment duration in the context of dosage [13,44]. Waterschoot et al. concluded that “on the basis of current literature, it is unknown how many hours, months, or weeks are needed to achieve the best effects” [13]. Using a meta-epidemiological approach, the meta-analysis by Dragioti et al. [44] examined the influence of treatment duration in the context of dosage, in patients with non-specific chronic low back pain, and they concluded likewise that treatment duration had no overall influence on the reported effects. However, in subgroup analyses, larger effect sizes for pain and disability emerged in favor of a long program with non-daily contact, while for quality of life a shorter duration was favorable. On the contrary, our results were consistent across outcomes, with the significant but nevertheless clinically negligible effect favoring moderate program for SF-36 physical health. Both of these SRs based their evidence synthesis on available literature. It is believed that our results add to this existing evidence by using large sample, pragmatic-register based primary data. Adding to this, we were able to use a controlled study design.

Previous preliminary reports on Swedish IMPR have indicated in various ways that treatment duration is an important factor for treatment outcome over time, which is in line with social cognitive theory that states “behavioral change takes time.” From interview studies with healthcare professionals, in primary care settings, it was reported that IMPR was useful but some professionals were frustrated by the short allowable IMPR program period of 6–8 weeks, stipulated as a result of the rehabilitation warranty—and felt it was too short a time to result in behavioral change [45]. However, short intensive interventions, even in the form of a few comprehensive assessment days, have been shown to have a positive effect, primarily on pain variables, and when these were followed by a four-week IMPR program, the positive effects also included several other factors such as pain interference with daily living, mood self-control, i.e., further psychosocial variables [46]. Another study reported that some patients may benefit from less intense rehabilitation interventions, such as a two-day interdisciplinary team assessment and the agreement on a rehabilitation plan with follow-up in primary care, and positive results on pain and somatic health remained at one-year follow-up [47].

The health care system and the provision of IMPR programs in Sweden are guided by national recommendations. It is therefore likely that most rehabilitation settings comply with these, which is reflected in some similarities in minimum time frames. We are not aware of any formal guidelines for the systematic allocation of different program durations and intensities; however, we cannot preclude that informal guidelines exist based on empirical evidence that would enable some specific selection. This, however, would only be possible in larger cities where multiple IMPR units are available. The shortest program duration measured in this study was four weeks of five full days each week, hence even the short duration treatment programs reached a minimum of 100 h (often referred to as a delimitation for more intensive programs). Whether an interaction effect exists between duration time in weeks and days per weeks, and how this impacts dosage/intensity, and whether these fit different patient characteristics needs further study. As relevant for the present analyses, however, days per week were adjusted for in all our between-group GEE-analyses.

We used a pragmatic register-based controlled study, which is a powerful method when RCTs are not possible. It has indeed been suggested that randomized trials suffer less from bias towards positive effects, but that systematic reviews including non-randomized but contemporaneous controls show similar effect estimates than those of randomized trials. There were nevertheless no relevant effects between our groups. Hence, we achieved for the first time, to our knowledge, a large controlled study with the potential to add to the discussion on optimal treatment duration forward.

### 4.4. Methodological Discussion

The present cohort was considered to be a representative chronic musculoskeletal pain sample, with similar demographics to patients normally participating in IMPR programs both in Sweden and other Western countries as presented in other studies [8,9,10,41]. Since the present study targeted patients in specialist care, the external relevance extends to patients in specialist care units rather than to subjects seen in primary care.

The motivation and theory for emphasizing treatment duration as a between-group factor—and not just building up a mathematical treatment index (e.g., treatment weeks × treatment days ×* treatment time)—is that we hypothesized that it takes time for behavior change to be learned and absorbed into individuals’ daily routines, as stated in social cognitive theory [14]. Hence, practicing and incorporating learned strategies from the IMPR into everyday life is likely to carry on after the intervention has ended. How these processes may have influenced long-term follow-up on our IMPR outcomes is unknown.

This study is the first of its kind to compare different durations of pain rehabilitation programs using large-scale longitudinal registry data. A possible limitation is that we don’t know the specifics of what is provided by the clinics, whether there are any possible systematic differences between short and longer programs, other than days/week, which we adjusted for in our analyses.

We used MCID as cut off-criteria for clinical change to indicate improvement and deterioration. However, the use of MCID does not in itself indicate whether an individual shifted from a clinically significant to a non-clinically significant level (or vice-versa). Differentiating further between various degrees of clinically significant change might have added more nuances to our understanding of acquired treatment effects from IMPR of different duration.

By using nationwide registry data, the study was executed cost-effectively without adding an extra burden to care providers. Our analyses were based only on the SQRP mandatory set of outcome measures, reported from all clinics linked with the registry. Other measures of equally high relevance, for instance, more recent and relating more specifically to constructs targeted within CBT- or ACT-based programs are optional and therefore not available for the full cohort. Still, our included measures reflect largely on the biopsychosocial understanding of chronic pain.

In our cohort, approximately 78% and 55% answered the post-intervention and the 12-month follow up, respectively, which is similar to other reports from the SQRP. Although higher attrition was found, at post-intervention, for the moderate IMPR—group there were no differences between those lost to follow-up compared to those completing data, irrespective of group allocation.

Our study represents real-world practice with a consecutive non-selective flow of patients, and no extra “RCT resources” or specialists, commonly trained in such trials were used. Our comparison groups were not randomized, and although our GEE analysis allowed controlling for between-group differences at baseline, unknown potential confounding has not been controlled for.

### 4.5. Implications and Future Directions

More systematic detailing of IMPR program content and praxis regarding ‘what and how’ is warranted for future comparisons, and team collaborative aspects should also be taken into account.

About 20% of patients reported a deterioration at follow-up. Further research presenting bidirectional data, presenting and further analyzing both positive and negative MCID, is warranted to increase knowledge of who will improve and who will not. Since baseline status is somewhat correlated with improvement, often in the direction that lower function will improve more, it will be especially important to perform further subgroup analyses, where interaction with treatment duration can also be taken into account.

Future register-based studies that aim to target different treatment approaches should consider the possibility of applying a large-scale register-based RCT design, to better target causality without increasing costs.

## 5. Conclusions

In conclusion, the majority of patients reported improvement after IMPR, indicating IMPR programs are effective across a biopsychosocial specter. There was, however, also a considerable proportion that deteriorated, which necessitates further prognostic study that includes personal as well as treatment characteristics. Despite statistical differences favoring moderate-duration IMPR programs over short-duration programs, the difference had no clinical significance. The IMPR content and team collaborative aspects can be highlighted in future pain rehabilitation research.

## Figures and Tables

**Table 1:** Patient baseline characteristics (*n* = 2698).

	Short IMPR (*n* = 924)	Moderate IMPR (*n* = 1379)	Long IMPR (*n* = 395)
Gender (%) females	78.1	81.4	84.1
Age mean (SD)	43.2 (10.5)	39.7 (10.7)	42.6 (10.0)
Education level (%)			
Elementary	13.7	12.0	12.2
Secondary	56.7	56.8	53.7
University	22.9	25.0	25.3
Other unspecified	6.4	5.1	7.8
Country of origin (%)			
Sweden	86.0	77.0	84.1
Other European country	7.5	7.2	5.6
Outside Europe	6.4	15.7	10.1
Pain duration (months) mean (SD)	110.0 (102.5)	93.7 (99.1)	119.8 (108.1)
Pain duration (months) median (IQR)	73.0 (137.4)	56.6 (122.9)	87.0 (145.1)
Number of pain regions mean (SD)	16.0 (8.4)	15.8 (8.6)	16.1 (8.4)
Working (%)	51.5	59.5	53.5

IMPR = Interdisciplinary multimodal pain rehabilitation, short IMPR: 4–9 weeks, moderate IMPR: 10 weeks, long IMPR: 11–18 weeks, SD = standard deviation

**Table 2:** Within-group results at baseline and after completion of an interdisciplinary multimodal pain rehabilitation (IMPR) program, and at 12-month follow-up (FU).

					Mean Within-Subject Change and Effect Size				
	Baseline Mean (SD)	Post-IMPR Mean (SD)	12-Month FU Mean (SD)	Baseline to PostIMPR *	95% CI	ES	Baseline to 12 Month FU *	95% CI	ES
SF-36 PCS (0–100)	28.5 (7.8)	30.8 (8.7)	31.7 (9.6)	2.0	1.7–2.3	0.28	2.8	2.4–3.2	0.37
SF-36 MCS (0–100)	34.6 (12.7)	39.1 (13.0)	38.7 (13.3)	4.3	3.8–4.9	0.35	3.8	3.1–4.4	0.32
Pain intensity last 7 days (NRS) (0–10)	7.0 (1.6)	6.1 (1.9)	6.0 (2.1)	−0.8	−0.9–−0.7	−0.51	−0.9	1.0–−0.8	−0.54
EQ-5D index (−0.594–1)	0.24 (0.30)	0.36 (0.32)	0.40 (0.34)	0.11	0.10–0.12	0.39	0.14	0.12–0.15	0.50
HADS A (0–21)	9.5 (4.8)	8.0 (4.5)	7.9 (4.8)	−1.4	−1.6–−1.2	−0.32	−1.3	−1.5–−1.1	−0.33
HADS D (0–21)	8.8 (4.4)	6.6 (4.2)	7.1 (4.6)	−2.2	−2.3–−2.0	−0.51	−1.6	−1.8–1.4	−0.38
MPI Impairment (0–6)	4.0 (0.8)	3.4 (0.9)	3.4 (1.1)	−0.5	−0.6–−0.5	−0.70	−0.5	−0.6–−0.5	−0.62
MPI Social Support (0–6)	3.5 (1.0)	3.4 (1.0)	3.4 (1.0)	−0.1	−0.1–0.0	0.10	−0.1	−0.2–−0.1	−0.10

* All parameters reached significance level <0.01, FU = Follow-up, SF 36 = 36-Item Short Form Survey, MCS = Mental Component Summary, PCS = Physical Component Summary, NRS = Numeric Rating Scale, EQ-5D = EuroQol five dimension scale, HADS A = Hospital Anxiety and Depression Scale (Anxiety), HADS D = Hospital Anxiety and Depression Scale (Depression), MPI = Multidimensional Pain Inventory. SD = Standard deviation, 95% CI = 95% Confidence Interval, ES = Effect size.

**Table 3:** Between-group results at baseline and after completion of IMPR program, and at 12-month follow-up. Marginal effects and p-values from pairwise comparisons with reference (short duration) are presented.

	Baseline		Post-IMPR				12-Month Follow-Up			
	Mean	95% CI	Mean	95% CI	*p*-Value	ES	Mean	95% CI	*p*-Value	ES
SF-36 PCS (0–100)										
short	27.9	27.4–28.4	30.2	29.7–30.8	Ref.		30.4	29.9–31.0	Ref.	
medium	28.6	28.2–29.1	31.6	31.1–32.2	<0.01	0.17	32.1	31.6–32.6	<0.01	0.26
long	29.3	28.5–30.1	29.7	28.8–30.6	0.335	−0.06	30.2	29.4–31.0	0.563	−0.03
SF-36 MCS (0–100)										
short	36.2	35.3–37.0	39.6	38.8–40.5			39.5	38.7–40.3		
medium	33.8	33.1–34.5	38.6	37.9–39.4	0.084	−0.08	38.5	37.8–39.2	0.055	−0.11
long	33.7	32.4–35.0	39.5	38.1–40.8	0.853	−0.01	39.2	38.0–40.4	0.672	−0.03
Pain intensity last 7 days (NRS) (0-10)										
short	7.0	6.9–7.1	6.2	6.1–6.3			6.1	6.0–6.3		
medium	7.0	6.9–7.1	6.1	5.96–6.2	0.166	−0.07	6.0	5.9–6.1	0.100	−0.08
long	6.8	6.7–7.0	6.2	5.9–6.4	0.891	−0.01	6.2	6.0–6.4	0.769	0.03
EQ-5D index (−0.594–1)										
short	0.25	0.23–0.27	0.35	0.33–0.38			0.36	0.34–0.38		
medium	0.22	0.20–0.23	0.37	0.35–0.39	0.313	0.05	0.39	0.37–0.41	0.052	0.11
long	0.28	0.25–0.31	0.34	0.31–0.38	0.632	−0.03	0.36	0.33–0.39	0.773	−0.02
HADS A (0–21)										
short	8.8	8.5–9.1	7.9	7.6–8.2			7.9	7.7–8.2		
medium	9.9	9.6–10.1	8.0	7.8–8.3	0.528	0.03	8.0	7.7–8.2	0.824	0.01
long	9.6	9.1–10.1	7.9	7.5–8.3	0.941	−0.01	7.8	7.5–8.2	0.650	−0.03
HADS A (0–21)										
short	8.5	8.2–8.8	6.5	6.2–6.8			6.8	6.6–7.1		
medium	9.0	8.8–9.2	6.6	6.4–6.8	0.519	0.03	6.7	6.5–6.9	0.555	−0.03
long	8.9	8.5- 9.3	6.7	6.4–7.1	0.293	0.07	7.0	6.6–7.4	0.425	0.06
MPI Impairment (0–6)										
short	3.9	3.9–4.0	3.4	3.4–3.5			3.4	3.4–3.5		
medium	4.1	4.0–4.1	3.4	3.4–3.5	0.547	0.02	3.4	3.3–3.5	0.240	−0.05
long	3.9	3.8–4.0	3.5	3.4–3.6	0.298	0.07	3.5	3.3–3.6	0.731	0.03
MPI Social Support (0–6)										
short	3.5	3.5–3.6	3.5	3.4–3.5			3.5	3.4–3.5		
medium	3.5	3.5–3.6	3.4	3.3–3.4	0.006	−0.11	3.4	3.3–3.4	0.003	−0.15
long	3.4	3.3–3.5	3.5	3.4–3.6	0.388	0.06	3.5	3.4–3.6	0.879	0.02

SF 36 = 36-Item Short Form Survey, MCS = Mental Component Summary, PCS = Physical Component Summary, NRS = Numeric Rating Scale, EQ-5D = EuroQol five dimension scale, HADS A = Hospital Anxiety and Depression Scale (Anxiety), HADS D = Hospital Anxiety and Depression Scale (Depression), MPI = Multidimensional Pain Inventory. 95%CI = 95% Confidence Interval, ES = Effect size.
